# QChIPat: a quantitative method to identify distinct binding patterns for two biological ChIP-seq samples in different experimental conditions

**DOI:** 10.1186/1471-2164-14-S8-S3

**Published:** 2013-12-09

**Authors:** Bin Liu, Jimmy Yi, Aishwarya SV, Xun Lan, Yilin Ma, Tim HM Huang, Gustavo Leone, Victor X Jin

**Affiliations:** 1 Department of Biomedical Informatics, The Ohio State University, Columbus, OH 43210, USA; 2 School of Computer Science and Technology, Harbin Institute of Technology Shenzhen Graduate School, Shenzhen, Guangdong, 518055, P.R., China; 3 Key Laboratory of Network Oriented Intelligent Computation, Harbin Institute of Technology Shenzhen Graduate School, Shenzhen, Guangdong, 518055, China; 4 Shanghai Key Laboratory of Intelligent Information Processing, Shanghai, P. R., China; 5 Department of Molecular Medicine, University of Texas Health Science Center at San Antonio, San Antonio, TX 78229, USA; 6 Comprenhensive Cancer Center, The Ohio State University, Columbus, OH 43210, USA

## Abstract

**Background:**

Many computational programs have been developed to identify enriched regions for a single biological ChIP-seq sample. Given that many biological questions are often asked to compare the difference between two different conditions, it is important to develop new programs that address the comparison of two biological ChIP-seq samples. Despite several programs designed to address this question, these programs suffer from some drawbacks, such as inability to distinguish whether the identified differential enriched regions are indeed significantly enriched, lack of distinguishing binding patterns, and neglect of the normalization between samples.

**Results:**

In this study, we developed a novel quantitative method for comparing two biological ChIP-seq samples, called QChIPat. Our method employs a new global normalization method: nonparametric empirical Bayes (NEB) correction normalization, utilizes pre-defined enriched regions identified from single-sample peak calling programs, uses statistical methods to define differential enriched regions, then defines binding (histone modification) pattern information for those differential enriched regions. Our program was tested on a benchmark data: histone modifications data used by ChIPDiffs. It was then applied on two study cases: one to identify differential histone modification sites for ChIP-seq of H3K27me3 and H3K9me2 data in AKT1-transfected MCF10A cells; the other to identify differential binding sites for ChIP-seq of TCF7L2 data in MCF7 and PANC1 cells.

**Conclusions:**

Several advantages of our program include: 1) it considers a control (or input) experiment; 2) it incorporates a novel global normalization strategy: nonparametric empirical Bayes correction normalization; 3) it provides the binding pattern information among different enriched regions. QChIPat is implemented in R, Perl and C++, and has been tested under Linux. The R package is available at http://motif.bmi.ohio-state.edu/QChIPat.

## Background

ChIP-seq (chromatin immunoprecipitation (ChIP) coupling with DNA sequencing) is widely used to precisely map the location of transcription factor (TF) binding or histone modification sites at a genome-wide scale [1-5]. Other related sequencing-based techniques DNase-seq [6], FAIRE-seq [6] are often used to define the open chromatin regions and identify accessible regulatory regions. Many computational programs [4,7-9] have been developed to identify enriched regions (referring to either binding sites or histone modification sites) for the ChIP-seq data. However, a majority of these programs were developed for a single ChIP-seq sample data. Given that many biological questions are often asked to compare the enriched regions under two different conditions, such as before and after using drugs, with and without chemical or hormone treatment, binding patterns of different transcription factors, or one transcription factor binding information in two cell types, it is critical to develop new programs to meet this need and to quantitatively compare two biological ChIP-seq samples. In our recent study [10], we found following TGFβ stimulation of the A2780 epithelial ovarian cancer cell line, ChIP-seq identified SMAD4 binding loci were classified into four distinct binding patterns: 1) Basal; 2) Shifted; 3) Stimulated Only; 4) Unstimulated Only, indicating that TGFβ stimulation alters SMAD4 binding patterns in epithelial ovarian cancer cells. However, the binding patterns were measured on a qualitative basis. Therefore, it is essential to develop a quantitative method to address this increasingly generalized biological question.

A study from Xu et al [11] employed a Hidden Markov Model (HMM) to identify the differential binding enrichments from two ChIP-seq samples (ChIPDiff), which was tested on three histone modifications data in two different cell types. However, the ChIPDiff program suffers from some shortcomings. Firstly, the HMM-based algorithm itself cannot distinguish whether those identified differential enriched regions are indeed significantly enriched or not. This is because in many cases a more enriched region in sample A comparing to sample B isn't necessarily an enriched region in sample A itself and vice versa. Therefore, the program tends to detect many false positive modification sites. Secondly, this program directly uses the samples' sequencing reads to perform the comparison without considering the information in the control experiment of each sample. However, as noted by Zhang et al. [9], the control sample is critical for modeling the sequencing and mapping biases. Thirdly, it is questionable whether this program is suitable for narrow peaks comparison since the program sets the bin size at least 1 kb. For example, the peak width for a typical TF binding site is around 300-500 bp. Fourthly, it fails to define a binding pattern for each identified differential binding site after comparing two samples. A few other studies have made the same efforts to develop programs, DBChIP [12], DIME [13], to detect differential binding sites in two samples, but these programs suffer from the same drawbacks either lacking distinguishing binding patterns or neglecting the normalization between samples.

In this study, we developed a novel quantitative method for comparing two biological ChIP-seq samples, called QChIPat, not only to detect the differential binding sites but also to classify them into distinct binding patterns. Our method employs a new global normalization method (nonparametric empirical Bayes correction normalization) [14-17], utilizes pre-defined enriched regions identified from single-sample enriched regions identification programs, uses statistical methods to define differential enriched regions, then defines binding pattern information for those differential enriched regions. Our program was tested on a benchmark data, histone modifications data [18] used by ChIPDiffs [11]. It was then applied on two study cases: one to identify differential histone modification sites for ChIP-seq of H3K27me3 and H3K9me2 data in AKT1-transfected MCF10A cells; the other to identify differential binding sites for ChIP-seq of TCF7L2 data in MCF7 and PANC1 cells.

## Results

### Evaluation of QChIPat with benchmark data

A benchmark testing data, the ChIP-seq of histone modification data from Mikkelsen et al [18], including H3K27me3 (K27), H3K4me3 (K4) and H3K36me3 (K36), was used for evaluating our program. The reason for choosing this data for the evaluation is the following: 1) it was used by ChIPDiff [11] and is easily compared to our program; and 2) differential histone modification sites have been validated in the same study [18]. A summary of the results for comparing different methods are shown in Table 1. We found that compared with ChIPDiff, QChIPat identified fewer differential histone modification sites (DHMSs). Two possible reasons may account for this: 1) the peak calling program BELT used in QChIPat identified a more stringent set of initial enriched regions compared with the one in ChIPDiff; and 2) the Wilcoxon rank test used in QChIPat removed less significantly different enriched regions. In order to minimize the bias from peak calling programs, other programs MACS [9] and FindPeaks [7] were also used for a comparison (Additional file 1 - Supplemental Table S1). Both programs identified even fewer DHMSs than BELT did. In addition, BELT was able to detect more DHMSs with "Only" patterns and "Shift" patterns compared with MACS and FindPeaks.

**Table 1:** A summary of comparing for the benchmark data with a bin size of 30 bp.

Methods	H3K4me3		H3K36me3		H3K27me3	
	**ESC enriched**	**NPC enriched**	**ESC enriched**	**NPC enriched**	**ESC enriched**	**NPC enriched**

Linear normalization	5738 (87.9%)*	913 (61.4%)	779 (29.9%)	1403 (58.7%)	3483 (77.2%)	495 (50.9%)
NEB normalization	6912 (78.2%)	**621 (71.3%)**	951(25.6%)	**1080 (70.8%)**	3664 (72.3%)	**428 (55.1%)**
Quantile normalization	**3918 (98.3%)**	2611 (31.3%)	**526 (43.3%)**	1952 (35.9%)	**2875 ( 85.8%)**	793 (38.8%)
Non-normalization	6959 (78.2%)	680 (70.3%)	741 (31.4%)	1409 (58.5%)	3266 (79.7%)	519 (49.3%)
ChIPDiff	12975	1767	1157	1227	3832	888

*Values in parentheses are percentage of DHMSs overlapping with ChIPDiff.

### Comparison of different normalization methods

ChIP-seq samples performed in different conditions and time usually have different numbers of raw sequencing reads due to the number of sequencing lanes conducted for each sample (sequencing depth) and multiple-matched or non-matched matched reads in the sample. Before comparing two samples, it is necessary to normalize the read counts to eliminate system errors and minimize sequencing depth difference between two samples. In our program, we employed three normalization methods including linear normalization, nonparametric empirical Bayes correction normalization and quantile normalization, which are based on different assumptions of the data distribution. A comparative summary of the three different normalization methods is shown in Table 1. The results of the method without using any normalization methods were also reported. The results of ChIPDiff were used as a baseline to gauge the performance of each different normalization method. For each method, the percentage of DHMSs overlapping with ChIPDiff results is shown in parentheses. The results showed that the DHMSs identified by quantile and NEB normalization methods highly overlap with those predicted by ChIPDiff, while the linear normalization method and non-normalization method have fewer overlapping DHMSs with ChIPDiff. For all the three data, the overlapping percentage between quantile and ChIPDiff is the highest one among all the three normalization methods in ESC, while NEB method achieves the highest overlapping percentage in NPC. Furthermore, the NEB method identifies more DHMSs than the quantile method in ESC and a similar number of DHMSs as that of the quantile method in NPC. Therefore, the overall performance of the NEB is better than that of other normalization methods on the three data. The results are not surprising since NEB normalization has two advantages compared with other normalization methods. Firstly, NEB estimates the proportion of the reads as *p*_0_=*n*_1_/*S*, where *n*_1 _is the frequency of the read with count 1 and *S *is the sequencing depth of the ChIP-Seq data. Secondly, NEB adjusts read counts based on both the observed read counts and the nature of the frequency distribution of the read counts.

### Optimization of the NEB-based method

**Figure 1:**
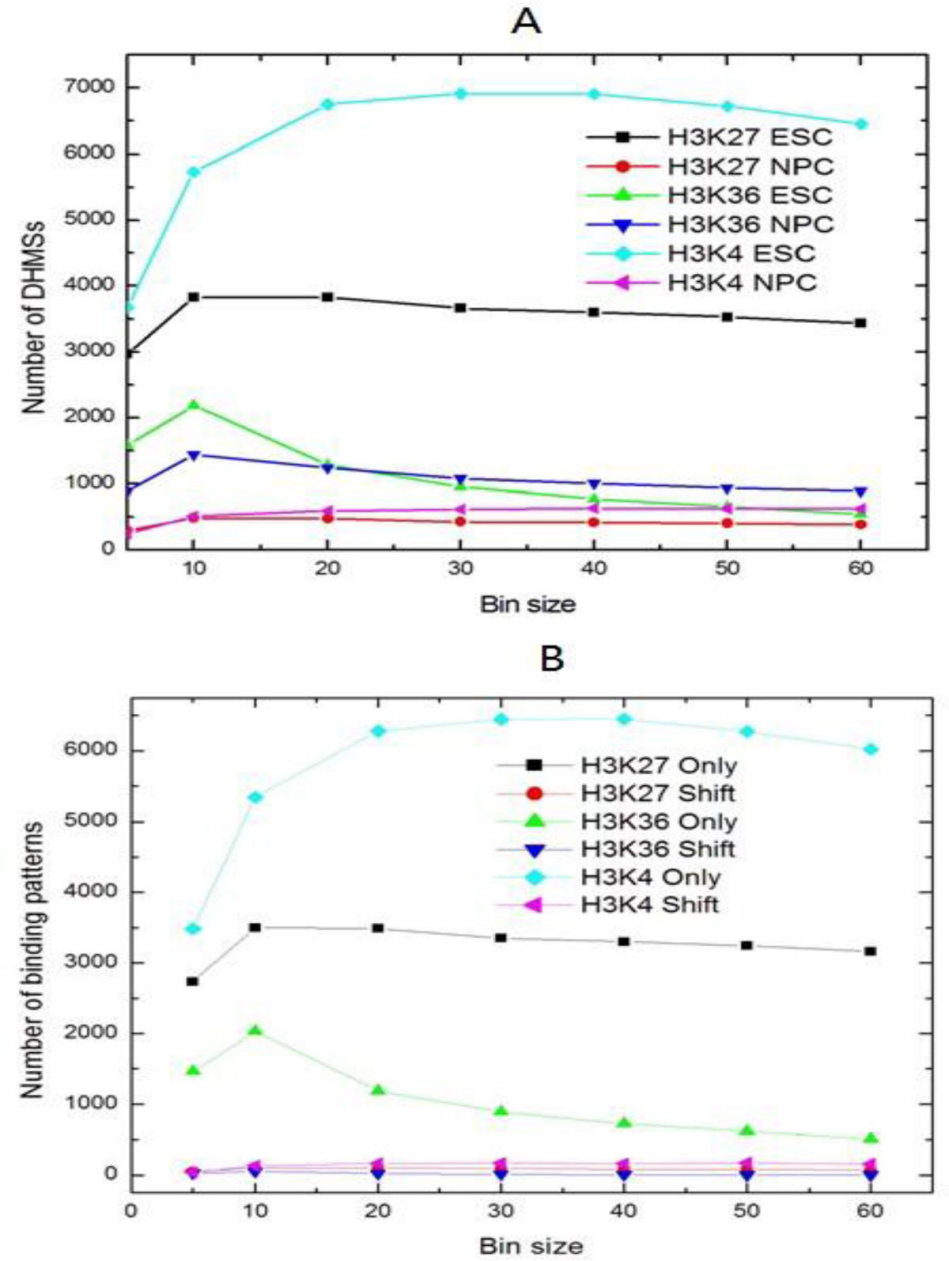
**The optimization of NEB-based method**. (A) The total number of DHMSs detected by the NEB normalization method with varying bin sizes. (B) The number of binding patterns identified with varying bin sizes.

In order to further optimize the NEB-based method, we tested the bin size parameter used in Wilcoxon rank test in our QChIPat program, to determine how this parameter would affect the number of the enriched regions and the types of binding patterns identified by our program. To optimize bin size and determine how its variance would affect the results, we first tested a range of different bin sizes from 5 to 60 bp for each bin, in which each enriched region was divided into equal length bins (see Methods). We found that the number of DHMSs for each sample didn't significantly change with different bin size tested (Figure 1A) when the NEB normalization method was used. However, when the bin size is higher than 10 bp, the number of identified DHMSs is very similar, suggesting that the optimal bin size for the NEB method may be between 20 and 40 bp. The binding pattern results show the same trend. The number of differential binding patterns remained stable when the bin size is higher than 10 bp (Figure 1B).

### Application to K9 and K27 datasets

To further test the performance and efficiency of QChIPat, we applied it to a study case, ChIP-seq of H3K27me3 and H3K9me2 in AKT1-tranfected MCF10A vs vehicle control, conducted in our laboratory. Many studies have shown that among many types of histone modifications, H3K27me3 and H3K9me2, two repressive marks are critical for normal and aberrant differentiation of stem and progenitor cells [19] as well as in the development of cancers [20]. Our previous study also demonstrated that epigenetic silencing of a set of AKT1-mediated genes [21]. AKT1 kinase is a key downstream effector of the phosphoinositide 3-kinase (PI3K) signaling pathway that regulates diverse cellular functions, including growth, proliferation, survival, metabolism, motility, angiogenesis, and vesicle trafficking [22,23]. Therefore, it is important to determine AKT1-mediated genome-wide histone modification patterns for H3K27me3 and H3K9me2 and its subsequent influence on downstream target genes. We chose the NEB normalization method since it performed best on the benchmark data H3K27me3, H3K4me3 and H3K36me3 compared to other three methods (see section Comparison of different normalization methods).

**Figure 2:**
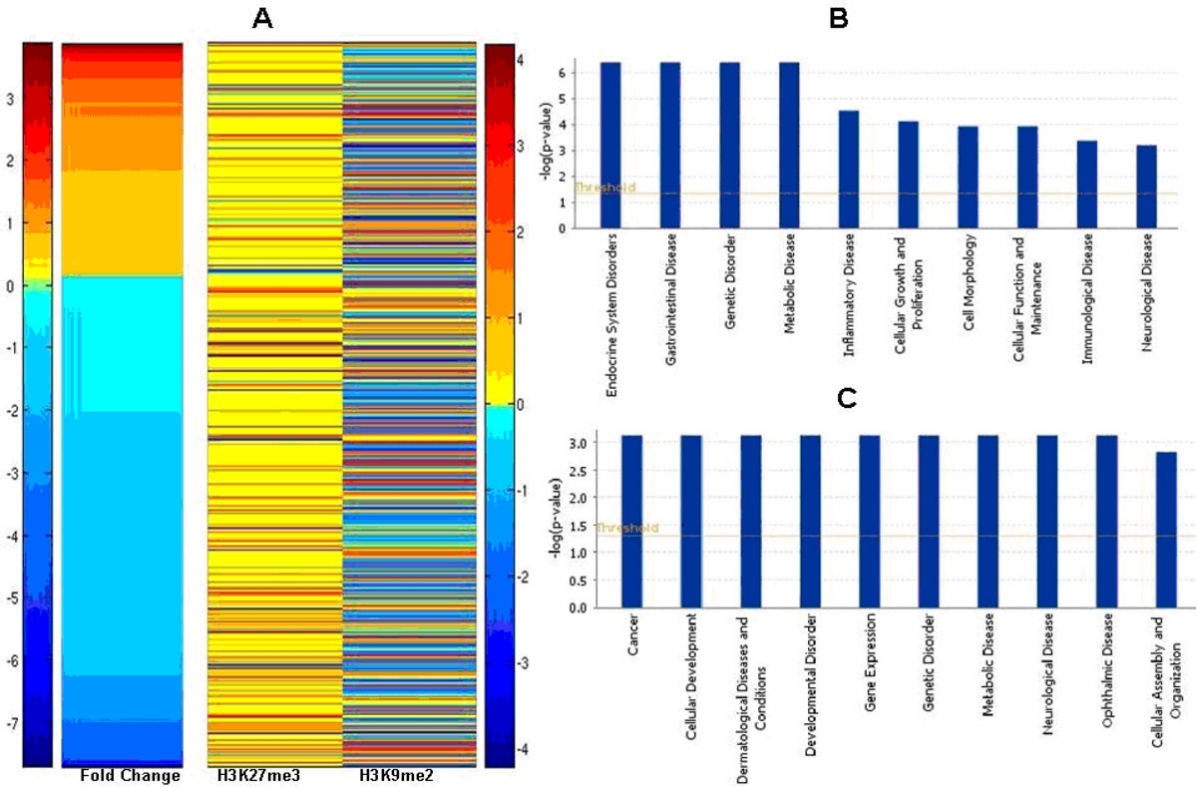
**Application of QChIPat on K27 and K9 datasets**. (A) Heatmap showing AKT1-mediated differential genes with enriched K27 and K9 marks. (B) GO analysis on common genes with both K9 and K27 marks showing high enrichment in the categories of tissue specific cellular functions. (C) GO analysis on common genes that lack either of two histone marks showing enriched in cancer related genes.

We have identified a total of 10,067 DHMSs for H3K27me3 in AKT1-transfected MCF10A cells. Of these, a majority of them (80.7%) were classified into "Only" or "Shift" binding patterns for AKT1-tranfected MCF10A cells. For H3K9me2 mark, of 2,532 DHMSs in AKT1-transfected MCF10A cells, 87.9% were either "Only" or "Shift" binding patterns (Table 2). The detailed information of the DHMSs is in Additional file 2, 3. Our results suggests that AKT1 signalling may trigger the switching of H3K27me3 or H3K9me2 modification sites, i.e. modifying different sets of nucleosomes on the genome, resulting in marking a totally different set of target genes in the case of "Only" binding. We further correlated the genes with DHMSs with a set of 2,893 AKT1-mediated differentially expressed genes in MCF10A cells, found that 661 genes have at least one DHMS for H3K27me3 or H3K9me2 marks (Figure 2A). Interestingly, we found that the DHMSs for H3K27me3 generally showed higher enrichment than those of H3K9me2. However, there was no significant difference in DHMSs' enrichments for AKT1-mediated up- or down-regulated genes. We further performed GO analysis on the sets of genes enriched for both H3K27me3 and H3K9me2 marks (Figure 2B and 2C). We defined two kinds of enriched genes: positive and negative enriched genes. Positive enriched genes are genes that are more enriched with either of two histone marks compared to the vehicle control (Figure 2B, common positive enriched genes between two datasets K9 and K27); while negative enriched genes less enriched with neither marks than the vehicle control (Figure 2C, common negative enriched genes between two datasets K9 and K27). Interestingly, the positive genes that are enriched in both the histone marks are highly enriched in the categories of tissue specific cellular functions, such as endocrine system disorders, gastrointestinal disease (Figure 2B). In contrast, the common negative enriched genes which lack these two histone marks are more enriched in cancer related genes (Figure 2C). The function of both groups of genes are epigenetically silenced by the presence of these histone modifications, while cancer related genes might be activated due to the loss of the epigenetic effect, which is consistent with the stem cell-like characteristics of tumour cells [24-26]. Next, we wanted to examine if there is functional difference between these two sets of genes that are marked by the two different histone modifications. Not surprisingly, genes which are positive with either mark in the AKT1-transfected cells show more tissue specificity. However, genes lacking H3K27me3 in AKT1-transfected cells are realted to cell death whereas the genes without H3K9me2 are more enriched in cancer genes.

**Table 2:** The binding patterns identified for the K9 and K27 datasets.

Sample (MCF10A)	Num. of DHMSs	Only	Shift
H3K27me3 (AKT1-transfected)	10,067	6,799	1333
H3K27me3 (vehicle control)	1,126	463	438
H3K9m2 (AKT1-transfected)	2,532	1346	879
H3K9m2 (vehicle control)	6,508	4507	1155

### Application to TCF7L2 in MCF7 and PANC1 cells

**Figure 3:**
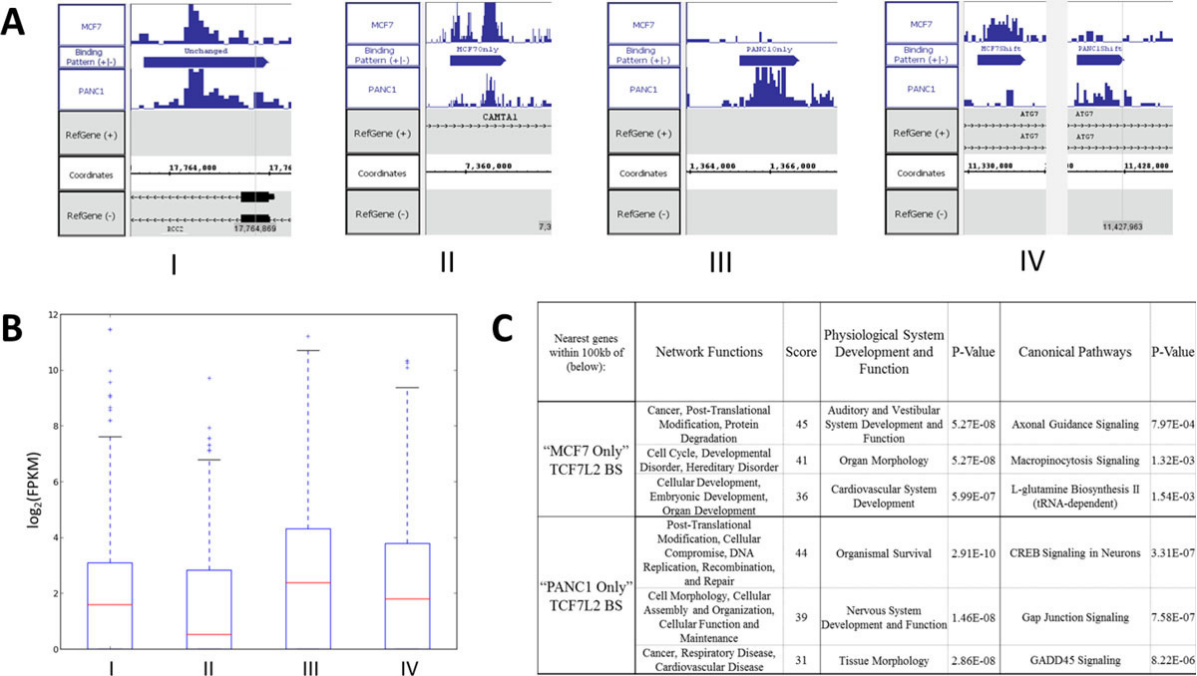
**Application of QChIPat to TCF7L2 datasets**. (A) Visualizations of read counts per bin and genomic locations of various different TCF7L2 binding patterns in MCF7 and PANC1. (B) Four boxplots of log_2_(FPKM) gene expression values. Before taking the log_2 _of the FPKM values, FPKMs equal to zero were removed and FPKMs between 0 and 1 were changed to 1. I) The boxplot of the MCF7 log_2_(FPKM) gene expression values for the genes associated with "MCF7 Only" TCF7L2 binding sites. II) The boxplot of the PANC1 log_2_(FPKM) gene expression values for the genes associated with "MCF7 Only" TCF7L2 binding sites. III) The boxplot of the PANC1 log_2_(FPKM) gene expression values for the genes associated with "PANC1 Only" TCF7L2 binding sites. IV) The boxplot of the MCF7 log_2_(FPKM) gene expression values for the genes associated with "PANC1 Only" TCF7L2 binding sites. (C) IPA analysis of genes associated with TCF7L2 "MCF7 Only" and "PANC1 Only" binding sites respectively.

In order to evaluate the performance of QChIPat on a particular transcription factor, we further tested QChIPat on ChIP-seq of the TCF7L2 in MCF7 and PANC1 cells. Using a set of parameters in which a bin size was 30 bp, p-value was less than 0.05, ratio was 1.2, difference was 0.8, BELT threshold was 0.91 for TCF7L2 in MCF7, and BELT threshold was 0.92 for TCF7L2 in PANC1, QChIPat identified 1,838 "MCF7 Only," 89 "MCF7 Shift," 27,163 "MCF7 Unchanged," 1,341 "PANC1 Only," 91 "PANC1 Shift," and 24,882 "PANC1 Unchanged" binding sites. Screenshots of examples for each type of binding pattern are shown in Figure 3A. We then examined the accuracy by correlating those "Only" binding pattern with their associated genes' expression levels. As expected, we found that genes associated TCF7L2 "MCF7 Only" have higher expression values in MCF7 cells than in PANC1 cells while genes associated TCF7L2 "PANC1 Only" have higher expression values in PANC1 cells than in MCF7 cells (Figure 3B). This result strongly demonstrated the accuracy of QChIPat.

We next performed IPA analysis on genes associated with MCF7 and PANC1 "Only" TCF7L2 binding sites (Figure 3C). Interestingly, we found that the genes associated with "MCF7 Only" binding sites differed significantly from the genes associated with "PANC1 Only" binding sites in terms of the three IPA categories of network functions, physiological system development and function, and canonical pathways. For all three gene expression categories, only cancer and post-translational modification (network functions) showed up for both cases of genes. This functional difference between genes associated with "MCF7 Only" TCF7L2 binding sites compared to genes associated with "PANC1 Only" TCF7L2 binding sites is expected, because the genes are differentially regulated by definition of the "Only" binding pattern: genes associated with "Only" differential enriched regions (TCF7L2 binding sites in this case) in one sample are not associated with any differential enriched regions in the other sample, Therefore, the significant difference in function of the two groups of genes supports the accuracy of QChIPat's "Only" pattern detection.

## Discussion and conclusions

In this study, we developed a quantitative method, QChIPat, to identify distinct binding patterns for two biological ChIP-seq samples, and the program is implemented in R, Perl and C++, and run in Linux system. Using a benchmarking test data, histone modifications data [18], we compared the performance of three different normalization methods on identification of DHMSs, including linear normalization, NEB correction normalization and quantile normalization, then compared it with ChIPDiffs program [11]. Although each normalization method has its own advantages, we found that NEB correction normalization is a more suitable normalization method in our QChIPat. Two notable advantages of NEB correction normalization are: 1) NEB estimates the proportion of the reads using the frequency of the read against the sequencing depth, which avoids both underestimating the proportion of highly abundant reads and overestimating the proportion of low and intermediate abundance reads, and 2) NEB adjusts read counts based on both the observed read counts and the nature of the frequency distribution of the read counts. This is able to correct abundant reads among high and low regions. Therefore, the enriched regions can be correctly identified. In addition, QChIPat has implemented a novel feature: categorizing binding (or modification) sites into three distinct binding patterns. This novel feature implementation is very important since quantitatively associating each of the differential binding sites with one of two samples is essential to reveal key biological functions. For example, Once we identify "Only" binding of genes in Sample A and Sample B, we are able to perform GO or other pathway analyses on these genes and may identify some interesting biological functions. This was demonstrated in our two study cases (Figures 2 and 3). Thus, compared to other similar programs, ChIPDiffs [11], DBChIP [12], and DIME [13], QChIPat is able to not only identify differential enriched binding regions between two samples but also to further classify these regions into distinct binding patterns associated with each sample.

Our QChIPat was further tested and validated on two study cases, ChIP-seq of H3K27me3 and H3K9me2 in AKT1-tranfected MCF10A vs. vehicle control, and ChIP-seq of TCF7L2 in MCF7 vs. PANC1 cells, both from our previous studies. Interestingly, both H3K37me3 and H3K9me2 marks showed two major binding patterns "Only" or "Shift" binding at AKT1-tranfected MCF10A cells (Table 2). Although our previous study has shown [21] AKT signaling can be a trigger of the epigenetic silencing at many downstream target genes through the cross-talk between DNA methylation and H3K27me3, it didn't show the relationship between H3K37me3 and H3K9me2. Nevertheless, it is a little bit surprising that both AKT1-mediated up- and down-regulated genes have at least one differential binding site for H3K27me3 or H3K9me2 marks but there is no significant difference in enrichments since both histone marks are recognized as repressive marks. It is possibly that activated AKT signaling may trigger downstream key transcription factors and further dynamically regulate epigenetic processes for the interplay of these two histone modifications. A more thorough underlying mechanism for this epigenetic process needs to be elucidated in an experimental study in the future. The test of QChIPat on ChIP-seq of TCF7L2 in MCF7 and PANC1 cells was used to evaluate its accuracy in detecting binding patterns of a particular transcription factor. The correlation between gene expression levels with the detected MCF7 and PANC1 "Only" TCF7L2 binding sites demonstrated that QChIPat is also applicable to compare narrow peaks in two ChIP-seq samples such as TFs in addition to broad peaks such as repressive histone modifications.

Several notable advantages of QChIPat include: 1) this software is able to make use of the information in control experiment; 2) this software incorporates a novel global normalization strategy: nonparametric empirical Bayes correction normalization; and 3) this software provides the binding pattern information among different enriched regions.

Nevertheless, there are several limitations. First, although three binding patterns were presented in this study, another binding pattern that may be interesting is where there is a sharp enriched region in one ChIP-seq sample, while a broad enriched region in the other ChIP-seq sample, but the total number of mapped reads are approximately the same. We look forward to deeper investigations on this binding pattern and will consider this pattern in the updated version of QChIPat in the future. Second, in this study, we only focus on comparing two biological ChIP-seq samples and identifying their differential binding patterns. Another interesting task is to identify differential binding patterns from two groups of ChIP-seq samples, which requires the comparison of more than two samples. We are studying new mathematical and statistical models for quantitatively identifying binding patterns from two groups of ChIP-seq samples in future work.

## Methods

### Program description

**Figure 4:**
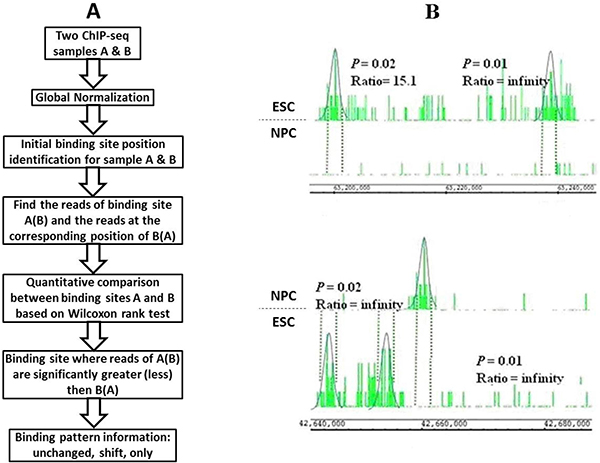
**Flowchart of QChIPat and an example**. (A) The flowchart of QChIPat depicts the steps to identify distinct binding patterns for two ChIP-seq samples. (B) This figure shows the comparison between two ChIP-seq samples: ESC H3K36me3 and NPC H3K36me3. The top subfigure displays two ESC "Only" H3K36me3 peaks compared zero NPC H3K36me3 peaks. The bottom subfigure shows an NPC "Shift/Unchanged" H36Kme3 peak compared with two ESC H3K36me3 peaks: one "Shift" (left) and the other "Unchanged" (right). The NPC H3K36me3 peak is labelled "Shift/Unchanged" because it corresponds both as a "Shift" peak in comparison with the left-hand ESC H3K36me3 peak and as an "Unchanged' peak in comparison with the right-hand ESC H3K36me3 peak.

The flowchart of the quantitative method QChIPat is shown in Figure 4A. Given two biological ChIP-seq samples, the first step is to normalize the two sample data in order to eliminate system errors and minimize sequencing depth difference. Three different statistical normalization methods are incorporated into the program, including linear normalization [27], nonparametric empirical Bayes correction normalization (NEB) [14] and quantile normalization [28]. The second step is to identify enriched regions by peak-calling programs. Three programs, including BELT1.0 [29] developed in our laboratory, MACS1.4.0beta [9] and FindPeaks4.0 [7] being widely used in the community, are currently employed in our QChIPat program. The third step is to distinguish the significantly differential enriched regions (DERs) between two ChIP-seq samples by using Wilcoxon rank test. The last step is to classify these DERs into three distinct binding patterns based on annotated genes information. Three distinct binding patterns are defined in our program: 1) "Unchanged" binding; 2) "Shift" binding; and 3) "Only" binding (Figure 4B). The program is implemented in R. BELT1.0 (the default peak calling program in the R package) is a program previously developed by our group. The parameter precision is used to specify threshold percentage number, followed by doubles [0.5, 1), indicating the threshold percentage number that will be used to calculate the threshold enrichment level. This parameter can be used to adjust FDR. Usage of this program requires installation of Java. A detailed description of the selection of optimal parameters for these programs is in Additional file 1.

### Normalization methods

Three optional normalization methods are provided in QChIPat, including linear normalization, nonparametric empirical Bayes correction normalization and Quantile normalization, which are based on different assumptions of the data distribution.

1) Linear normalization [27]: Linear normalization is the simplest and most straightforward way to normalize ChIP-seq data by scaling the total number of reads of different ChIP-seq samples into the same level. This normalization method is reasonable if the total number of reads in the two samples is roughly the same. In our program, linear normalization is used, in which all reads in a sample is divided by the total number of reads in the sample so that the reads in two samples are in the same scale. This can be formulated as *p = x*/*n*, where *x *is the number of reads in each bin (in order to calculate the distribution of the data, the whole genome is divided into small fixed size regions which are called bins in this study, and then the total number of reads in each bin is counted), proportion *p *is the normalized reads number in a given bin and *n *is the total number of reads of the ChIP-Seq data.

2) Nonparametric empirical Bayes correction normalization (NEB) [14]: The drawback of the linear normalization is that the proportion *p *of the missing reads (because the sequencing depth is not enough, some reads would be missing) from the data is assigned to be zero, which underestimates the *p *of highly abundant reads and overestimates the *p *of low and intermediate abundance reads. Therefore, the nonparametric empirical Bayes correction normalization (NEB) is used to solve this problem. The nonparametric empirical Bayes correction normalization has been successfully applied to normalize the different SAGE libraries with different sequencing depth [9]. In this study, the Good-Turing estimator (SGT) [30] is used to implement the empirical Bayes. Given a sample with total number of reads of *S*, the aim of empirical Bayes estimation is to estimate the true proportion of read *i *(*P_i_*) from the data. The observed read count *c *is estimated as:

(1) $$c^{*}=\frac{cn_{c+1}+n_{c+1}}{n_{c}}$$

Where *n_c _*is the number of reads with count *c*. The proportion of undetected reads *P*_0 _is estimated as:

(2) $${p_{0}}^{}=\frac{n_{1}}{S}$$

Where *S *is the sequencing depth (*S = n*_1_+*n*_2_+...). The empirical Bayes estimator for proportion of a read with count c (P_c_*) is renormalized as:

(3) $$p_{c}^{*}=\frac{n^{*}-p_{0}n^{*}}{S^{*}}$$

Where S* is the corrected total read count after SGT:

(4) $$S^{*}=\sum n_{c}c^{*}$$

3) Quantile normalization [28]: If the distribution of the two samples is assumed to be the same, quantile normalization can be applied to the ChIP-seq data for further comparison. In this package, the read number distribution of the first sample is used as the reference distribution and the second sample is transformed to make sure that the distribution of the two samples is the same. The transformation can be formulated as ${\text{x}}_{\text{norm},\text{2}}=F_{1}^{-1}\left(F_{2}\left(x\right)\right).F_{\text{1}}$ is the distribution of the first sample and *F*_2 _is the distribution of the second sample. Since the Wilcoxon rank test only makes use of the rank of the read number, choosing either the first or second sample does not affect the final comparison results.

### Comparison of enriched regions

In order to reduce the number of false positive enriched regions and make use of the samples obtained by control experiments, three optional publicly available enriched region identification programs are included in our method, including BELT [29], MACS [9] and FindPeaks [7]. The users are able to select one based on their datasets and interests. After identifying the enriched regions in each sample, each enriched region is divided into equal length bins. The number of bins in each enriched region is a parameter, which needs to be optimized based on the experiments. If the length of the enriched region is less than the bin number, the bin number is automatically set to the length of the enriched region. Since the length of enriched regions may be different from each other, the length of bin may vary for different peaks. After the normalized read number is counted, the number of reads in each bin in the corresponding location in the comparing sample is also calculated, and then the two groups of reads number can be used for Wilcoxon rank test.

### Wilcoxon rank test

There are two options of Wilcoxon rank tests: Wilcoxon signed rank test and Wilcoxon rank sum test. If it is believed that the read number of the two ChIP-Seq data is paired, Wilcoxon signed rank test should be used (wil.paired=TRUE). Let *Z_i _= X_i _*- *Y_i _*for *i *= 1, ... *b_n_*, and assume that the *Z_i _*is independent; each *Z_i _*comes from a same continuous population and is symmetric about its median *θ*. If not paired, Wilcoxon rank sum test should be used (*wil.pair*=FALSE). Let F denotes the distribution of X, the read enrichment of Sample A, and G denotes the distribution of Y, the read enrichment of Sample B. Assume that F and G have the same shape and they differ only by median: G(*t*)=F(*t-θ*) [31].

H_0_: θ=0. (The read enrichment difference between Sample A and B is not significant);

H_1_: θ>0 (Sample A has more significant read enrichment);

H_2_: θ<0 (Sample B has more significant read enrichment).

For signed rank test, let *R_i _*represents the rank of each ordered |*Z_i_*| and *φ_i _*denote whether *Z_i _*is positive. When *Z_i _*is positive, *φ_i _*is 1. Otherwise, it is -1. The Wilcoxon signed rank statistic T+ is calculated as $T^{+}=\sum_{i=1}^{bn}R_{i}\varphi_{i}$. For the rank sum test, all × and Y are put together and the pooled data is ranked. Let *S_j _*denotes the rank of *Y_s_*, and then the Wilcoxon rank sum statistics is the sum of all *S_j _*($W=\sum_{j=1}^{bn}S_{j}$). Then, by checking the corresponding table, p-value can be calculated.

### Determination of differential enriched regions

Once a p-value from Wilcoxon rank test and read ratio ($r=\sum_{i=1}^{bn}x_{1,i}^{}/\sum_{j=1}^{bn}x_{2,j}^{}$, *x*_1,*i *_stands for the reads number in each bin of sample A and *x*_2,*j *_stands for the reads number in each bin of sample B) and difference ($d=\left|\sum_{i=1}^{bn}x_{1,i}^{}-\sum_{j=1}^{bn}x_{2,j}^{}\right|$) have been calculated, a set of significant differential enriched regions in each sample are determined based on the user defined cutoffs.

### Definition of distinct binding patterns

Each differential enriched region, identified from comparison of a sample A with a sample B, is classified into one of the following three distinct binding patterns: 1) Sample A/B "Unchanged" - one of two corresponding differential enriched regions in separate samples that are associated with the same annotated gene and are within 1 kb distance of each other; 2) Sample A/B "Shift"- one of two corresponding differential enriched regions in separate samples that are associated with the same gene, but are more than 1 kb apart from one another; and 3) Sample A/B "Only" - a differential enriched region associated with a gene in one sample, but no differential enriched regions are associated with that gene in the other sample. Note that the program, by default, associates a gene with a differential enriched region if they are within 100 kb of each other.

### Benchmark data

The ChIP-seq of histone modification data from Mikkelsen et al [18] (the raw data can be downloaded at ftp://ftp.broad.mit.edu/pub/papers/chipseq/Mikkelsen2007/alignments/) including H3K27me3 (K27), H3K4me3 (K4) and H3K36me3 (K36) were used for the benchmarking test to evaluate the performance of our program QChIPat.

### Data output

QChIPat provides comprehensive output information including a summary report, differential enriched regions in each sample with BED format and UCSC web browser and Affymetrix Integrated Genome Browser compatible wiggle (.wig) for the visualization, as well as the assigned binding pattern of differential enriched regions.

### Data sets for two study cases

ChIP-seq of H3K27me3 and H3K9me2 in AKT1-tranfected MCF710A cells is obtained from our previous study [21]. The ChIP-seq of TCF7L2 in MCF7 and PANC1 cells were obtained from our previous study [32].

## Competing interests

The authors declare that they have no competing interests.

## Authors' contributions

VXJ, BL and JY conceived and coordinated the study. BL, JY, ASV, YM and XL developed the R package and BELT1.0; BL, JY and YM analysed the ChIP-seq data; and BL, JY, XL and VXJ drafted the manuscript and the content was approved by all authors.

## Supplementary Material

Additional file 1 Click here for file

Additional file 2 Click here for file

Additional file 3 **Table S1**. Number of DHMSs identified by different programs for identifying enriched regions. Click here for file

## Declarations

The publication costs for this article were funded by University of Texas Health Science Center at San Antonio.

This article has been published as part of *BMC Genomics *Volume 14 Supplement 8, 2013: Selected articles from the International Conference on Intelligent Biology and Medicine (ICIBM 2013): Genomics. The full contents of the supplement are available online at http://www.biomedcentral.com/bmcgenomics/supplements/14/S8.
