# Supplementary material for: QChIPat: a quantitative method to identify distinct binding patterns for two biological ChIP-seq samples in different experimental conditions
Source: BMC Genomics. 2013 Dec 9;14(Suppl 8):S3. doi: 10.1186/1471-2164-14-S8-S3 (PMC4042236; doi:10.1186/1471-2164-14-S8-S3)
Supplement: Additional file 1 [file 1471-2164-14-S8-S3-S1.PDF]

Supplementary Material

Program implementaion

The program is implemented in R. BELT1.0 (the default peak calling program in the R package) is a program previously developed by our group. The parameter precision is used to specify threshold percentage number, followed by doubles [0.5, 1), indicating the threshold percentage number that will be used to calculate the threshold enrichment level. This parameter can be used to adjust FDR. MACS 1.4.0 and MACS 2.0.4 with default parameters is used to find the enriched regions. In order to use this program as the enriched region finding software, installation of MACS is required, since R package cannot install MACS automatically. FindPeaks version 4.0 is the third optional enriched region finding software. The parameters are set as: -minimum 8; -no_peaks_header; peaklength is set fixed length as 300,-trim 0.2 -subpeaks 0.5 –duplicatefilter; Usage of this program requires installation of Java.

Availability and requirements

R (2.10.1 or higher version), Perl (5.12.1 or higher version) and Java environment (6 or higher version) are required before use. MACS need installation before use. Some data operation scripts are written in perl and C++, since they are more efficient in file operation than R.

Supplemental Tables

Supplemental Table S1. Number of DHMSs identified by different programs for identifying enriched regions.

Programs	ESC	NPC	ESC Only	ESC Shift	NPC Only	NPC Shift
FindPeaks	2534(83.5%)	374(45.7%)	2377	96	248	66
MACS	2534(83.5%)	374(45.7%)	2377	96	248	66
BELT	6912 (78.2%)	621 (71.3%)	6290	67	447	44
ChIPDiff	12976	1768	NA	NA	NA	NA

*Values in parentheses are percentage of DHMSs overlapping with ChIPDiff.

The program ChIPDiff is unable to analyze the binding patterns, so the binding patterns are not aviliable.
